# Endovascular repair of late type IIIb endoleak after endovascular aneurysm repair: a case report

**DOI:** 10.1186/s12872-019-1138-8

**Published:** 2019-08-01

**Authors:** Jiang Shao, Xin Zhang, Yu Chen, Yuehong Zheng, Bao Liu

**Affiliations:** Department of Vascular Surgery, Peking Union Medical College Hospital, Peking Union Medical College and Chinese Academy of Medical Sciences, Beijing, China

**Keywords:** Endoleak, Abdominal aortic aneurysm, Late type IIIb endoleak, Endovascular repair

## Abstract

**Background:**

Type IIIb endoleak is a rare complication after endovascular aneurysm repair (EVAR) and the understanding of type IIIb endoleak is insufficient.

**Case presentation:**

Three elderly patients with previously successfully performed EVARs were sent to our center due to abdominal pain and diagnosed with late type IIIb endoleak. The type IIIb endoleak fabric tears were closed with cuffs or stents by endovascular retreatment. The patients recovered adequately and were discharged from hospital.

**Conclusions:**

Endovascular repair with empirical relining could be a good choice for treatment of late type IIIb endoleak with good prognosis.

**Electronic supplementary material:**

The online version of this article (10.1186/s12872-019-1138-8) contains supplementary material, which is available to authorized users.

## Background

Endovascular aneurysm repair (EVAR) is the most common and the least invasive method to treat abdominal aortic aneurysms (AAAs) [1]. Short-term benefits of EVAR have been demonstrated, while some complications during long-term follow-up should be more recognized [2]. Endoleak is divided into five types and is a common complication after EVAR(some researchers consider endotension as type V endoleak but some think there are only four types) [3]. Thereinto type III endoleak is caused by defeat in the graft. In type IIIa endoleak, blood leaks from disconnection of modular overlap. Type IIIb endoleak arises from fabric defects and may cause high risk of aneurysm rupture. In an analysis of the EUROSTAR registry data, the rupture risks of patients with late type III endoleak were about nine times higher than those of other registry patients and were even higher than for those of patients with type I endoleak [4]. Despite the high risks, fortunately, type III is uncommon [5]. According to a study of 439 patients after EVAR, the incidence rate of type III endoleak is only 1.4% (*n* = 6) [6]. In another study, only two type IIIb endoleaks in 886 patients after EVAR and the incidence rate is 0.23% (2/886) [1]. In systematic review by Lowe et al., there were only 50 endoleak patients (including 10 cases of type IIIb) form 1998–2017 [2]. Therefore, the understanding of type IIIb endoleak is insufficient. We present three patients who had no abnormalities for years after previously performed EVAR until late type IIIb endoleak emerged.

## Case presentation

### Case 1

A 90-year-old man underwent successful EVAR with a body of stent (Talent 24 × 14 × 155 mm, Medtronic®) and an iliac limb (Talent 14 × 14 × 75 mm, Medtronic®) 4 years prior and attended routine follow-up from the third month after the procedure. The reports of computed tomography angiography (CTA) remained almost unchanged. Several vascular calcifications and noncalcified plaques with vascular stenosis appeared in both lower limbs with noncalcified plaques and moderate stenosis in the proximal segment of left renal artery. No increase of aneurysm size was reported.

The patient did not have apparent symptom for 4 years until the day he started to feel abdominal pain and had one episode of syncope. The patient was transported to our center and was diagnosed with impending rupture of AAA. After examination by abdominal CTA, it was thought to be a type I endoleak, meaning leakage from attachment sites.

His medical history was notable for hypertension for 20 years and smoked 20 cigarettes per day for over 50 years. On palpation, a pulsatile painless mass approximately 6.0 cm × 5.0 cm could be appreciated.

**Fig. 1:**
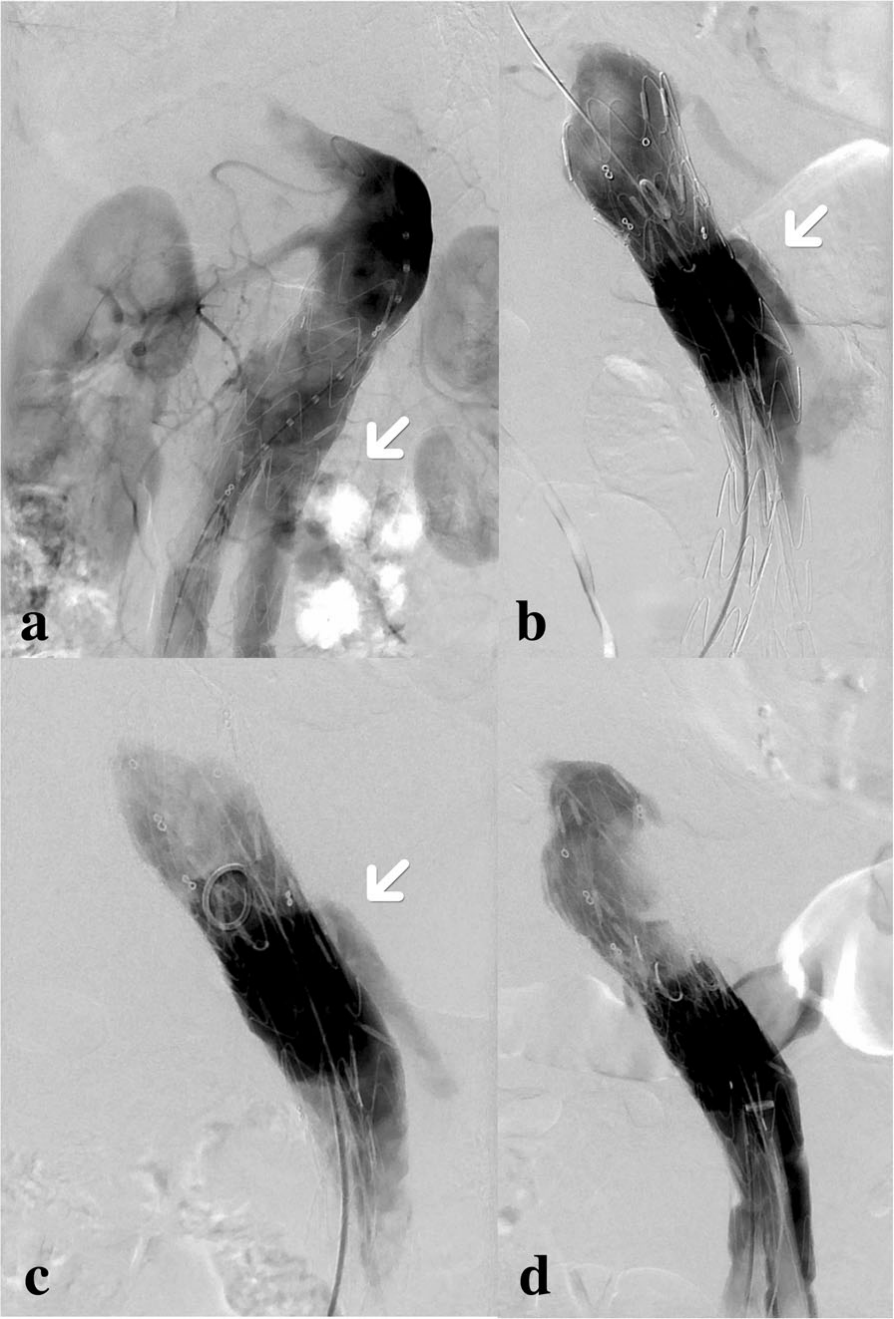
The procedures of endovascular retreatment were recorded. **a** An endoleak (arrow) can be seen below right renal artery; **b** RAO of angiography after the first cuff was released showing the type IIIb endoleak (arrow); **c** After the second cuff was released, the speed of endoleak (arrow) apparently slowed down (better seen in the supplementary materials); **d** After the third cuff was released, the endoleak stopped


At surgery, angiography clearly revealed that the neck of aneurysm was mildly dilated compared to 4 years prior. Lateral image suggested that the possibility of type I endoleak was large, while fabric tear of stent graft could not be excluded (Fig. 1a). A cuff (Ankura 34 × 34 × 40 mm, Lifetech®) was released through the left femoral artery below the right renal artery. The type I endoleak ceased. However, on RAO of angiogram, a fabric tear was found in the anterior wall of the stent, confirming type IIIb endoleak. (Fig. 1b, Additional file: 1). The secondary cuff (Ankura 34 × 34 × 60 mm, Lifetech®) was released below the right renal artery with its distal end above the iliac limbs. Subsequently, the speed of the endoleak was apparently slowed down (Fig. 1c, Additional file: 2). To absolutely eliminate the endoleak, a third cuff (Excluder 32 × 32 × 40 mm, Gore®) was released exactly to the fabric tear of the stent with a CODA balloon used to dilate both ends of the stent. Endoleak had disappeared on the final angiography (Fig. 1d). After the procedure, the patient’s recovery was satisfactory.

**Additional file 1:** Result after the first cuff deployed in case 1. Comparing Additional files 1 and 2, it can be found that the speed of the endoleak blood flow slowed down after the second cuff was deployed in case 1, which is not clear enough just through figures. (AVI 1630 kb)
**Additional file 2:** Result after the second cuff deployed in case 1. Comparing Additional files 1 and 2, it can be found that the speed of the endoleak blood flow slowed down after the second cuff was deployed in case 1, which is not clear enough just through figures. (AVI 1100 kb)


Follow-up was performed once a year and there is no recurrence of endoleak or related complication found in 4 years.

### Case 2

A 79-year-old woman who was hospitalized 38 months ago was diagnosed as having a pseudoaneurysm. A successful EVAR was performed with an Aegis bifurcated stent-graft system (Aegis-B (unibody stent) body: 24 × 80 mm, limbs: 12 × 40 mm, 12 × 30 mm, MicroPort®).

Approximately 38 months after the EVAR, the patient felt lower abdominal pain when blood pressure fluctuated to 160/90 mmHg. The pain radiated to the left hip and Visual analogue scale (VAS) score was approximately 6–7.

**Fig. 2:**
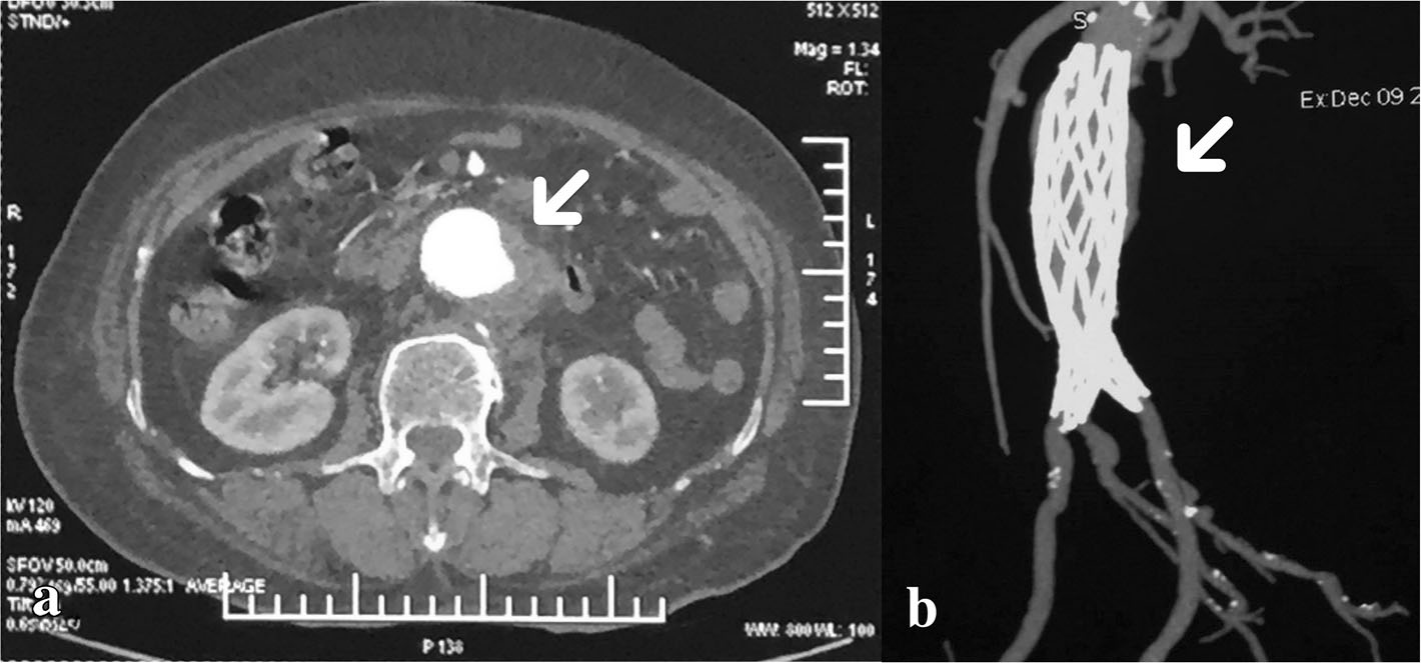
CT was taken before endovascular repair. The contrast medium spilled through the fabric tear in the left wall of middle segment of the stent (arrows both in **a** and **b**)

With mild symptoms for approximately 1 month, the symptoms progressed to severe with continuous cutting pain with sweating, malaise and occasional vomiting. VAS score was 10. The highest systolic pressure was 220 mmHg. Abdominopelvic enhanced CT in another hospital suggested that an abdominal aneurysm stretched from renal arteries to the bifurcation of iliac arteries and the diameter was approximately 3 cm. The endoleak was observed from the left wall of the middle segment of the stent (Fig. 2).

The past medical history was notable for hypertension and diabetes without smoking or alcohol use.

**Fig. 3:**
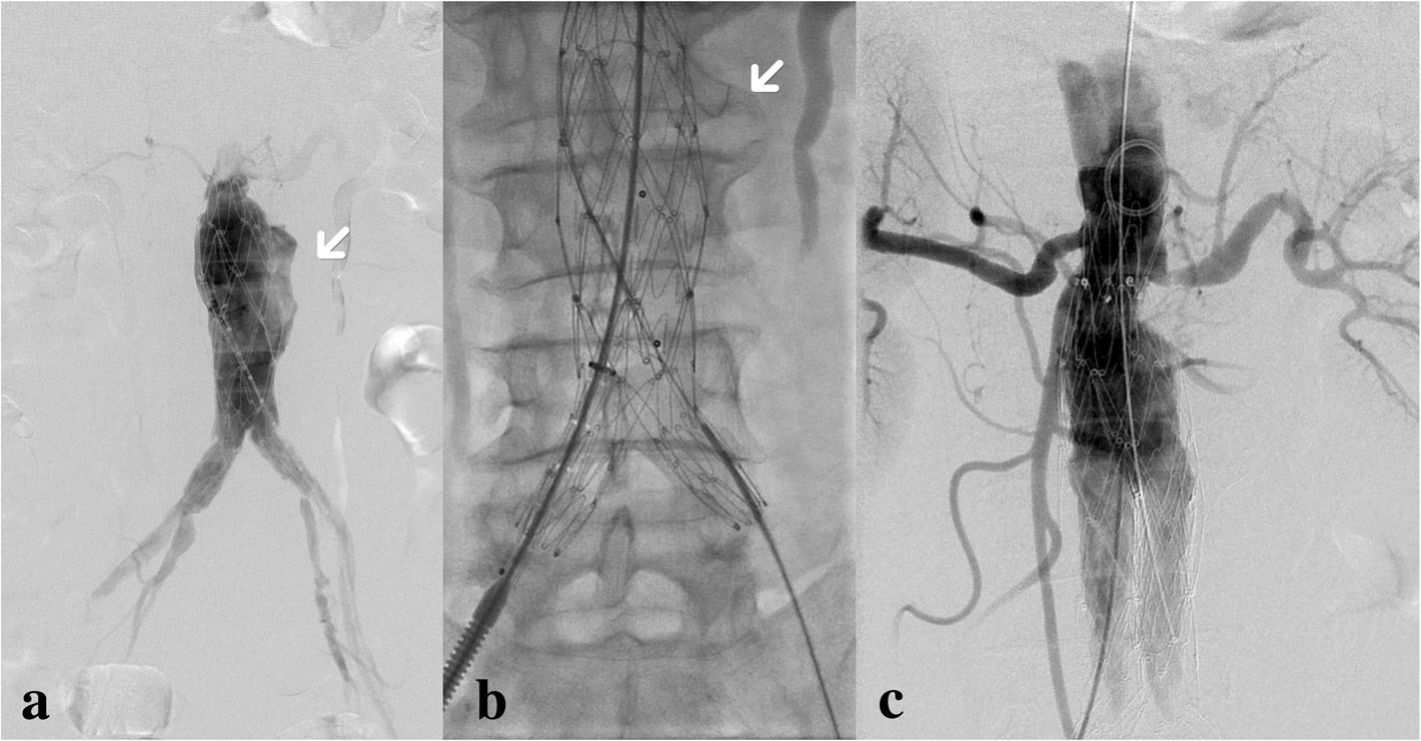
**a** The endoleak was seen from the left wall of main body of previous stent; **b** the guidewire passed through the fabric tear; **c** repair was finished with the main body of the stent and the left iliac limb

During surgery, after the catheter entered into the main body of the stent and we performed angiography, an endoleak was found from the film mulching area of the stent, meaning type IIIb endoleak (Fig. 3a). The fabric tear was confirmed with the guidewire able to pass through the lateral wall of stent (Fig. 3b). Endovascular retreatment was performed with the main body of stent graft system (Endurant II 25 × 16 × 145 mm, Medtronic®) released at the level of renal arteries and limbs (Endurant II 16 × 13 × 80 mm, Medtronic®) released at the bifurcation of left iliac arteries. After dilation with a CODA balloon, the endoleak disappeared (Fig. 3c). Postoperative recovery was satisfactory.

At a follow-up frequency of once a year, there is no recurrence of endoleak or related complication found in 2 years after the repair.

### Case 3

A 75-year-old man who was hospitalized to receive coronary artery bypass graft (CABG) 6 years prior was found to have AAA through CT during in-hospital stay and underwent EVAR in other hospital. And endoleak repair was performed once 3 years prior in the same hospital. He had progressive abdominal pain for 23 days and had been diagnosed with possible rupture of AAA through CTA in a local hospital (however, with no report or image seen by us) and was admitted to the emergency department of our center due to limited condition in that hospital. In his past medical history, the patient had hypertension up to 180/100 mmHg, coronary artery disease and myocardial infraction (inferior wall) without smoking or alcohol consumption.

On physical examination, a pulsatile mass with the diameter of 15 cm was found in the middle part of the upper abdomen.

**Fig. 4:**
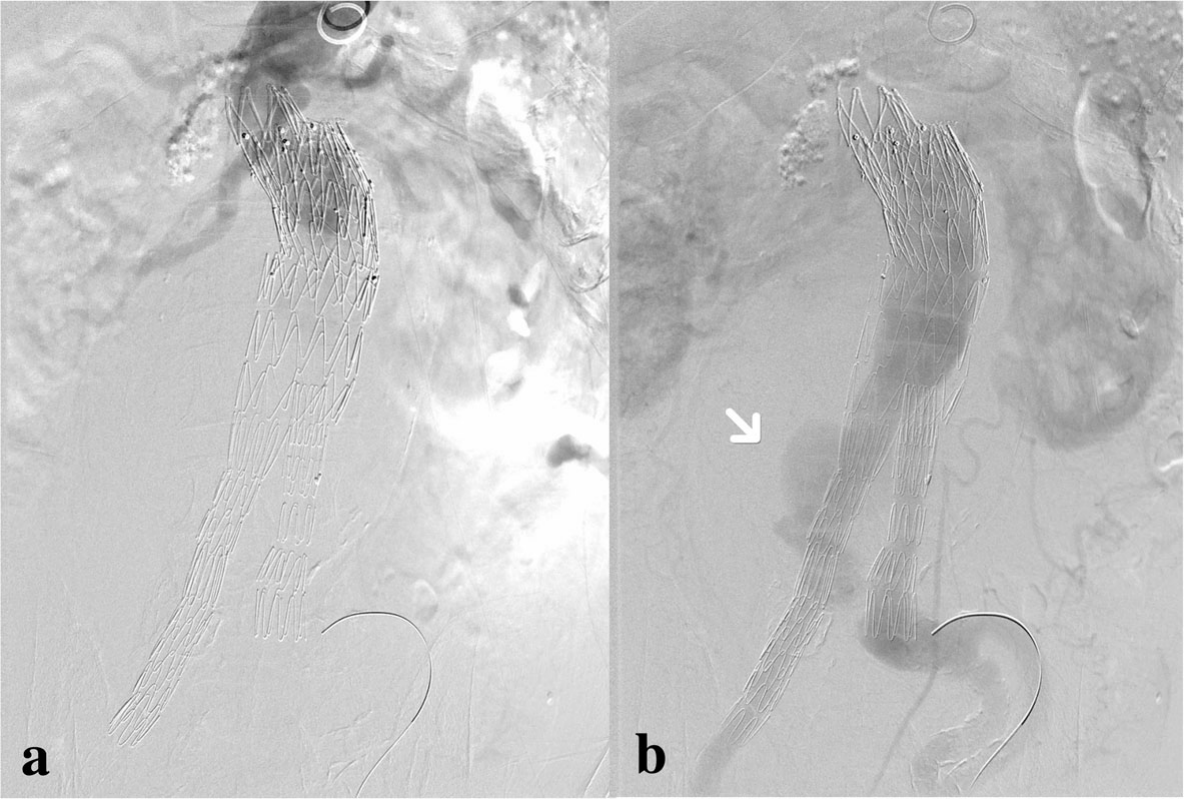
**a** This image was taken before contrast agent arrived at the open of stent; **b** contrast agent spilling out was seen (arrow)

**Fig. 5:**
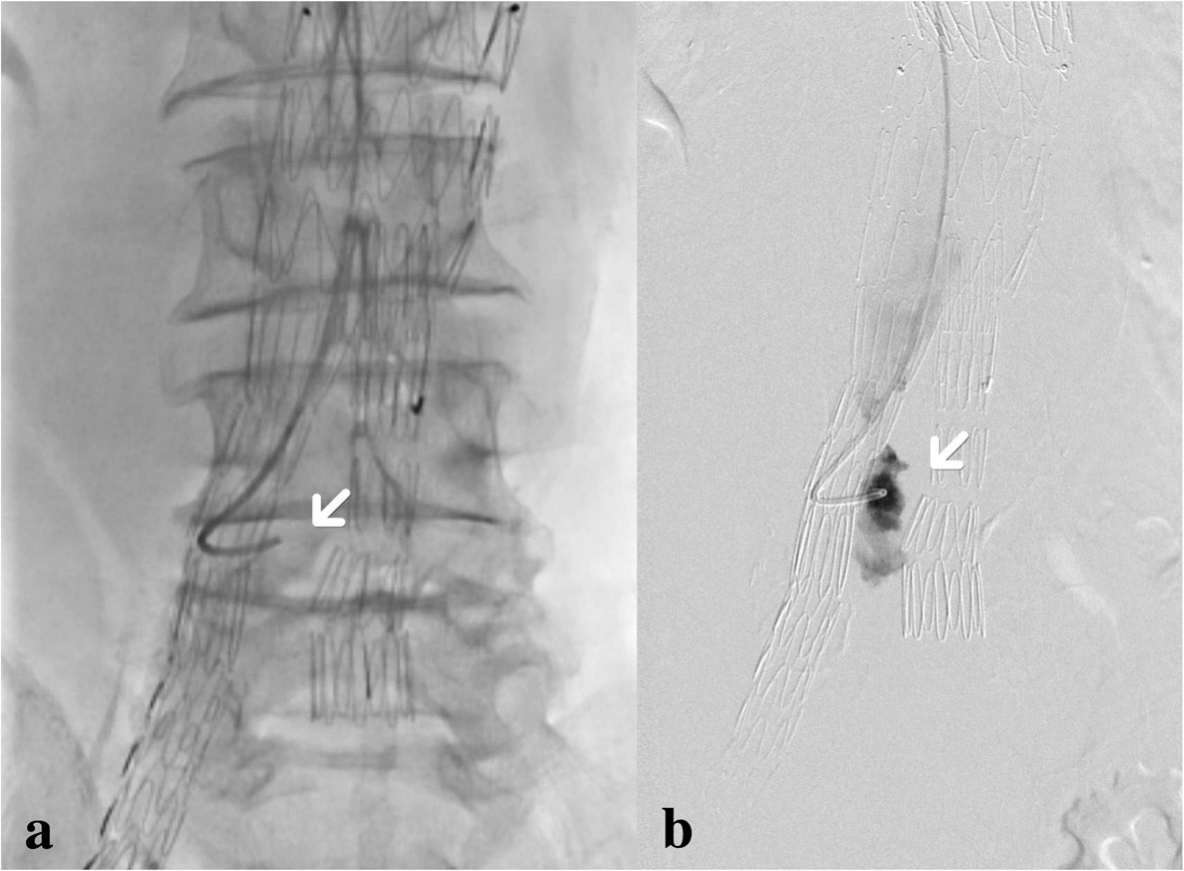
**a** We made the catheter pass through the wall of stent, directly confirming type IIIb endoleak; **b** contrast agent appeared out of the stent through the catheter

**Fig. 6:**
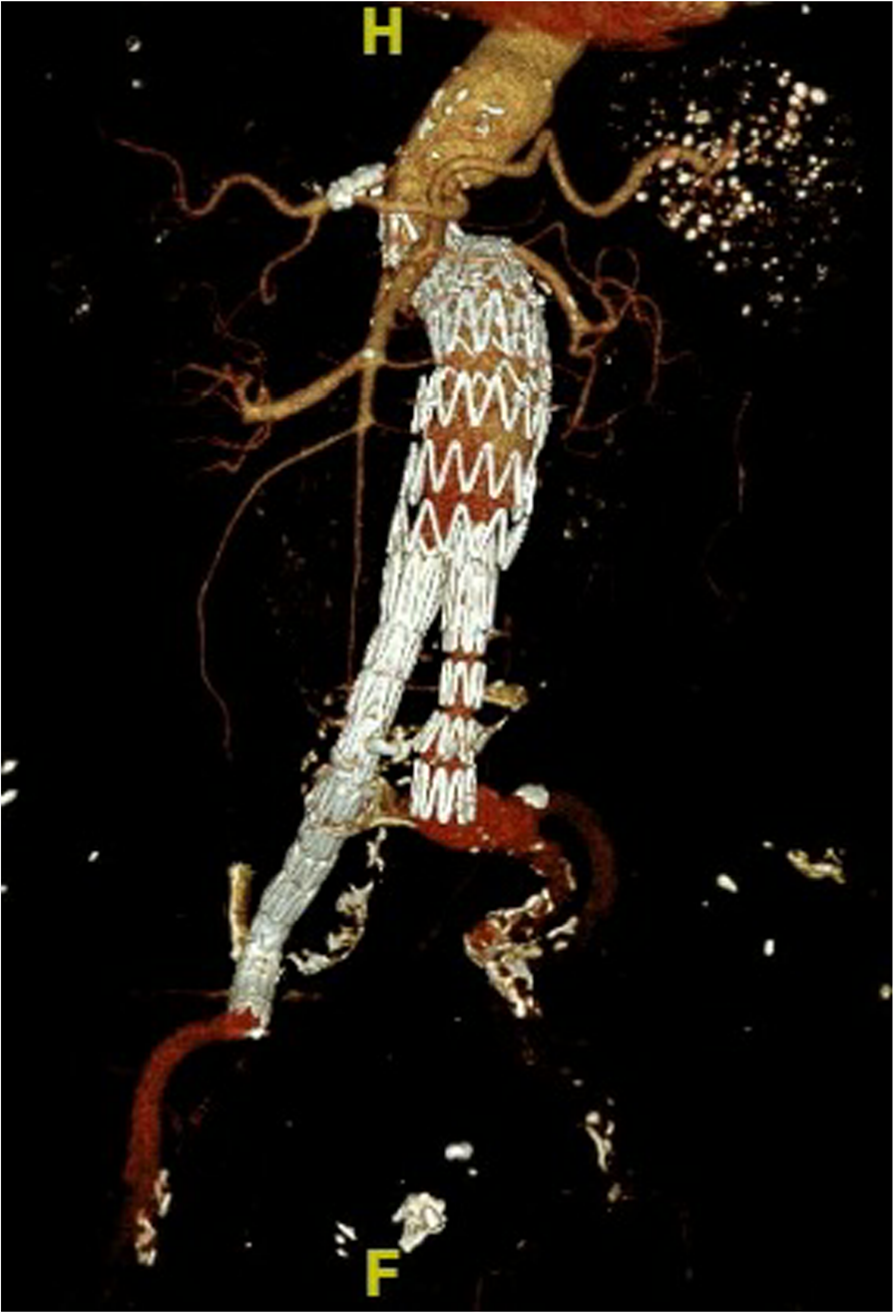
CTA was taken after the surgery but before discharging from hospital. Endoleak has disappeared

At surgery, a possible type IIIb endoleak was detected at the upper part of the right iliac limb (Fig. 4). A 5F catheter was passed through the wall of the iliac stent graft, confirming the fabric tear (Fig. 5). Then, another iliac limb (Excluder 12 × 12 × 100 mm, Gore®) was successfully deployed with the aneurysm sac embolization subsequently. After the procedure, CTA was taken (Fig. 6) and the patient’s recovery was satisfactory.

To date, no recurrence of endoleak or related complication was found in the regular follow-up which was performed once a year.

## Discussion and conclusion

With the incidence of type IIIb endoleak at only approximately 0.23% [1], another study including 701 patient samples indicated that type III occurred in 19/701, including 17/701 (2.4%) type IIIa and 2/701 (0.3%) type IIIb [7]. Thus, type IIIb endoleak is very rare and it is a rare opportunity to be able to report three cases of late type IIIb endoleak. To represent more clearly, the information of the three cases was summarized in Table 1.

**Table 1:** Summary of three patients

Patient	1	2	3
Gender&Age	90, Male	79, Famle	75, Male
Past History	Hypertension	Hypertension	Hypertension
	Smoking	Diabetes	post-CABG
			Smoking
			Endoleak repair once 3 years prior (detail unknown)
EVAR Stent	Talent, Medtronic; Body:24 × 14 × 155 mm, limb: 14 × 14 × 75 mm	Aegis-B, MicroPort; (unibody design) Body: 24 × 80 mm, limbs: 12 × 40 mm, 12 × 30 mm	Unknown
Years of Repair after EVAR	4	3	6
Repair Device	(1) Ankura 34 × 34 × 40 mm, Lifetech (2) Ankura 34 × 34 × 60 mm, Lifetech (3) Excluder 32 × 32 × 40 mm, Gore	Endurant II, Medtronic; Body: 25 × 16 × 145 mm, iliac limb: 16 × 13 × 80 mm	Excluder 12 × 12 × 100 mm, Gore
Follow-up	4 times/4 years	2 times/2 years	2 times/2 years
Recurrence or Related Complication	No	No	No

According to cases reported to date, the causes of late type III include spontaneous fabric break [8], stent fracture [9, 10] and stent migration caused by progression of disease [11]. Spontaneous break here means speculations. For instance, the fabric was folded when the stent was loaded

to the device system and was pressed against the metal strut [8]. In a study of 62 patients suffering from AAA, the patients were treated with the AneuRx stent graft system (Medtronic, Santa Rosa, Calif) and presented with broken attachment sutures and metal-ring fractures after EVAR (median, 5.1 years). As a result, metal-ring fractures were associated with 75% type III endoleak, with an adjusted RR of 13.9 (*P* = 0.003) [10]. Because stent migration easily lead to the interactions between metal part and fabric, stent migration is one of risk factors for type I and III endoleak [11]. In our case, no stent migration or apparent fracture was seen under CT or angiography. Spontaneous fabric break appears to be the most possible cause. However, spontaneous fabric breaks and very tiny stent fractures need be differentiated by postmortem examination. Thus, we can clearly diagnose late type IIIb and only assume the spontaneous fabric break to be the cause. However, we cannot confirm the exact cause for fabric tears in our cases.

When type IIIb endoleak is confirmed, it can be repaired by empirically relining the additional devices. If endovascular methods fail, surgical conversion would be the final option [12]. In the systematic review by Lowe et al. including 50 endoleak patients from 1998 to 2017, 69% were treated with endovascular procedures [2]. In a multicenter retrospective study by Geert et al. with 965 patients who underwent EVAR from 1995 until 2014, 88% of type III endoleaks (*n* = 22) were treated using endovascular techniques and 12% (*n* = 3) were treated with open surgical conversions. Nevertheless, recurrence remains possible. During the follow-up after initial type III endoleak endovascular treatment, endoleak recurred in 25% of patients, not significantly different with incidence rate after initial EVAR. By contrast, in the three patients with open surgical conversion of type III endoleak in the multicenter study by Geert et al., open surgical conversion was used to deal with severe situations. One was due to rupture and another was because of an aorto-duodenal fistula [1]. In our cases, we chose to use cuffs and stents to achieve endovascular retreatment and the prognosis of patients was satisfactory.

A multicenter retrospective study of 965 patients analyzed the relationship between stents of various generations and incidences of type III endoleak and found that incidence of first or second-generation was 12.7% and incidence of third generation decreased to 1.2%. Time to endoleak increased from 3.87 years (1^st^ or 2^nd^ generation) to 5.92 years (3rd generation) [1]. Apparent development of stents improved prognosis after EVAR and decreased type III endoleak, However, there remains no stent-graft system able to absolutely avoid type III endoleak; continuous modification is needed.

In conclusion, type III endoleak can appear at any period after EVAR and there may be no apparent symptoms. Therefore, long-term follow-up is needed with CTA or other imaging examinations. Even if there is no endoleak, any stent fracture or migration could be found. Although incidence of endoleak and other complications after EVAR has apparently decreased with the development of stent-graft systems, further improvement of stents is expected to achieve better prognoses for patients after EVAR. If, unfortunately, an endoleak is found, endovascular retreatment is a good choice with good prognosis; open surgical conversion can be a final option.

## Data Availability

The datasets generated and analyzed during the current study are available from the corresponding author on reasonable request.

## Abbreviations

AAA: Abdominal aortic aneurysm
CABG: Coronary artery bypass graft
CTA: Computed tomography angiography
EVAR: Endovascular aneurysm repair
VAS: Visual analogue scale

## References

[1] Maleux G, Poorteman L, Laenen A (2017). Incidence, etiology, and management of type III endoleak after endovascular aortic repair. J Vasc Surg.

[2] Lowe C, Hansrani V, Madan M, Antoniou GA. Systematic review of type IIIb endoleak after elective endovascular aneurysm repair. J Cardiovasc Surg. 2018. 10.23736/S0021-9509.18.10446-0. 10.23736/S0021-9509.18.10446-0 29616524

[3] Chaikof EL, Dalman RL, Eskandari MK (2018). The Society for Vascular Surgery practice guidelines on the care of patients with an abdominal aortic aneurysm[J]. J Vasc Surg.

[4] Harris PL, Vallabhaneni SR, Desgranges P (2000). Incidence and risk factors of late rupture, conversion, and death after endovascular repair of infrarenal aortic aneurysms: the EUROSTAR experience. European collaborators on stent/graft techniques for aortic aneurysm repair. J Vasc Surg.

[5] Antoniou GA, Georgiadis GS, Antoniou SA (2015). Late rupture of abdominal aortic aneurysm after previous endovascular repair: a systematic review and meta-analysis. J Endovasc Ther.

[6] Lal BK, Zhou W, Li Z (2015). Predictors and outcomes of endoleaks in the veterans affairs open versus endovascular repair (OVER) trial of abdominal aortic aneurysms. J Vasc Surg.

[7] Skibba AA, Evans JR, Greenfield DT (2015). Management of late main-body aortic endograft component uncoupling and type IIIa endoleak encountered with the Endologix Powerlink and AFX platforms. J Vasc Surg.

[8] Banno H, Morimae H, Ihara T, Kobayashi M, Yamamoto K, Komori K (2012). Late type III endoleak from fabric tears of a zenith stent graft: report of a case. Surg Today.

[9] Wanhainen A, Nyman R, Eriksson M, Björck M (2008). First report of a late type III endoleak from fabric tears of a zenith stent graft. J Vasc Surg.

[10] Ueda T, Takaoka H, Petrovitch I (2014). Detection of broken sutures and metal-ring fractures in AneuRx stent-grafts by using three-dimensional CT angiography after endovascular abdominal aortic aneurysm repair: association with late endoleak development and device migration. Radiology.

[11] Mezes P, Sallam M, Diamantopoulos A, Taylor P, Ahmed I (2014). Zenith cook limb type IIIB endoleak causing aneurysm rupture five years after EVAR. Vascular.

[12] Brown KE, Heyer KS, Matsumura JS (2008). Late type III endoleak and graft failure of an ancure stent-graft. J Vasc Interv Radiol.

